# From LMX to Individual Creativity: Interactive Effect of Engagement and Job Complexity

**DOI:** 10.3390/ijerph17082626

**Published:** 2020-04-11

**Authors:** Guadalupe Vila-Vázquez, Carmen Castro-Casal, Dolores Álvarez-Pérez

**Affiliations:** Department of Business Administration and Marketing, Universidade de Santiago de Compostela, Facultade de Ciencias Económicas e Empresariais, 15782 Santiago de Compostela, Spain; mdolores.alvarez@usc.es

**Keywords:** LMX, engagement, job complexity, creativity, high-tech and knowledge-based SMEs

## Abstract

This study explores how the quality of leader–member exchange (LMX) is associated with employees’ creativity via engagement, and the moderating role of a job characteristic related to knowledge (job complexity) in this relationship. A moderated mediation model was tested on a sample of 320 employees from emergent high-tech and knowledge-based small and medium enterprises (SMEs) in Spain. The results supported an indirect influence of LMX on individual creativity through engagement. Additionally, this study found that job complexity accentuated the impact of engagement on creativity. More importantly, the findings confirmed that the intensity of the LMX–engagement–creativity relationship was moderated by job complexity. That is, the relationship was stronger when job complexity was high. Taken together, this study improves the understanding of the factors that contribute to increased employee creativity, an important outcome for high-tech and knowledge-based SMEs.

## 1. Introduction

In the current knowledge economy, individual creativity (i.e., “the production of novel and useful ideas by an individual” [1] (p. 126)) has become crucial to firms [2,3,4]. As their context is characterized by strong competition and the scarcity of resources, small and medium enterprises (SMEs) increasingly depend on employees’ ideas for their longer-term survival and to achieve a competitive advantage [5]. Those firms operating in high-tech and knowledge-intensive industries particularly need to promote employee creativity as the foundation for the innovation and the introduction of new processes and products to the market [6,7]. 

In knowledge-intensive SMEs, employees are the main bearers of tacit knowledge and their competences and creativity are important for the innovation process [8,9]. Since these firms cannot compete in efficiency via economies of scale, they rely more on leveraging employee creativity [10].

Although creativity and innovation are conceptually different, they are closely related constructs [11,12]. Individual creativity focuses on the generation of new and useful ideas, while innovative behavior also encompasses their application [2,4,13]. Some scholars (e.g., Amabile [14], Janssen [15], and Scott and Bruce [16]) consider creativity to be the first phase of the innovation process, which also encompasses the promotion of ideas and their realization. In the same vein, LePine and Van Dyne ([17], p. 865) emphasized that "innovation begins with recognition and generation of novel ideas or solutions that challenge past practices and standard operating procedures”. According to Joo et al. ([18], p. 394), creativity is important in itself because it is “the seed of innovation”.

Research has extensively analyzed employee characteristics (such as creative self-efficacy, personality traits or creative skills, among others) as antecedents of individual creativity (see the narrative review by Anderson et al. [2], and the review by Cai et al. [11]). The importance of social context as a driver of individual creativity has also been recognized [2,11,12]. Empirical evidence has shown that, in general, leadership styles focused on the relationship, such as leader–member exchange, boost creativity ([4,19,20]; see also the meta-analytical reviews by Carnevale et al. [13], Hammond et al. [21], and Lee et al. [12]). The social exchange theory [22] and the principle of reciprocity [23] hold that when individuals perceive that they have benefited from a positive relationship, they will feel indebted and obliged to reciprocate it exhibiting positive behaviors. In a close and positive relationship between leader and follower, the latter will engage in creative behavior by receiving support, trust, and resources from the leader [12]. Nevertheless, it is necessary to go deeper into the underlying processes through which leader–member exchange (LMX) is associated with creativity. The fact that not all studies have supported this association (e.g., Clegg et al. [24]) and the high variability in the relationships found in meta-analytical reviews suggest the existence of potential mediators and moderators in this complex relationship. 

By proposing engagement as a mediator and job complexity as a moderating contextual variable, this paper helps to clarify the process model linking LMX to individual creativity and responds to the Qu et al.’s [25] recent call for new research that analyzes the moderating and mediating variables in the LMX-creativity relationship. 

Specifically, the goals of this study are (a) to examine employee engagement as a mechanism that transmits the effect of LMX on individual creativity, and (b) to analyze job complexity as a boundary condition that intensifies the effect of employee engagement on individual creativity.

As stated by Kahn [26], engagement is “the simultaneous employment and expression of a person’s “preferred self” in task behaviors that promote connections to work and to others, personal presence (physical, cognitive, and emotional) and active, full performances” (p. 700). The physical dimension of engagement is manifested in the energy and effort exerted in the work role, the cognitive dimension is manifested in the attention and absorption in the work role, and the emotional dimension is characterized by a positive affective response (enjoyment and activation) towards the work role. According to Rich et al. [27], this conceptualization of engagement goes beyond the traditional focus on physical or cognitive effort applied to a work role; it describes the degree to which employees simultaneously invest all their energies “in a holistic and connected manner” ([27], p. 618) into the performance of their work roles, and how intensely and persistently they apply them.

Although individuals make choices concerning investing themselves in and expressing themselves through work roles [26,27], leaders facilitate the conditions that render such actions possible [28]. Kahn and Heaphy [29] highlighted the relevance of the relational context, such as LMX, for engagement. Furthermore, it has been argued that employees engaged in their work roles tend to employ all their energies, display their real identity, thoughts, and feelings, and consequently, be more creative [26]. By analyzing the effect of engagement on individual creativity, this research addresses Anderson et al.’s [2] call for more research concerning motivational psychological states associated with individual creativity.

In the literature concerning creativity, intrinsic motivation (i.e., the desire to spend effort based on interest, pleasure, and enjoyment of the work itself [30,31]) has been considered a proximal antecedent of individual creativity [1,14], although a lesser effect than expected has been found (see Grant and Berry’s review [32]). The use of engagement as a mediating variable was based on its recognition as one of the main motivational constructs [27,33]. Although engagement shares conceptual space with intrinsic motivation (both involve effort and persistence; [27]), engagement extends further because it is not just about performing a task for the enjoyment and pleasure it entails, it also incorporates the simultaneous investment of cognitive and emotional energies [26,27,34]. In this regard, the study by Rich et al. [27] found that engagement and intrinsic motivation were different constructs and that engagement explained additional variance in job performance and organizational citizenship behavior. 

Despite growing evidence showing that engagement is a motivational psychological state relating contextual factors to employees’ attitudes and behaviors [27,35,36], there is a lack of studies examining their mediating role in the LMX–individual creativity relationship. To the best of the authors’ knowledge, only two studies [37,38], carried out in the service sector in India and South Korea, have empirically tested engagement as an intervening mechanism between LMX and innovative behavior. Since innovative behavior also covers the implementation of ideas, these studies do not allow to determine the differential contribution of LMX on creativity through engagement. Scholars [2,12] advocate the need to examine creativity separately. 

In addition, according to the interactionist perspective [39], creative behaviors arise from the interplay between individual and contextual factors. This study proposes that the effect of engagement on individual creativity will be conditioned by the opportunities offered by the task context (represented by job complexity). 

Job complexity is among the most important characteristics of contemporary jobs [40], particularly in knowledge-based firms. It is recognized that more complex jobs offer employees more opportunities to be creative [41,42,43]. Most of the creativity research has considered job complexity (usually measured as a combination of identity, variety, significance, autonomy, and feedback) as an antecedent of creativity [2]. However, so far, research examining the moderating role of job complexity is still limited.

Volmer et al. [19] claimed that “opportunities provided within high-quality LMX are best used in conjunction with job design features” (p. 475). Shalley et al.’s [44] study showed that job complexity interacts with growth need strength and a supportive work context to affect individual creativity. Audenaert et al. [45] found that job complexity positively moderated the influence of psychological empowerment on employee innovation. Recently, Cai et al. [46] confirmed that job characteristics strengthened the association between employee psychological capital and creativity. However, how job complexity performs as a moderator in the LMX–engagement–creativity relationship has remained unexplored. Therefore, this study is the first to analyze these relationships.

Our study extends the literature on the implications of relational leadership (in our case, LMX) on individual creativity. Although there is a growing body of research that has used the LMX framework to explain creativity, according to Tierney ([47], p. 175) “there is still much to be investigated in this realm”. 

First, this study responds to the call by Qu et al. [25] for further research on mediators in the LMX–individual creativity relationship. Specifically, engagement, a psychological motivational state that manifests itself in the physical, cognitive, and emotional energies exerted at work, is proposed. Although engagement is recognized as a robust motivational construct and calls for further research on its impact on creativity have been made (e.g., Anderson et al.’s [2]), little is yet known about its effect on creativity [48]. 

Second, given that the engagement–creativity link may be affected by the task context, we extend the research by analyzing the moderating role of job complexity, a key job characteristic in knowledge-intensive firms. Although both engagement and job complexity are relevant to creativity, surprisingly, their interactive effect has not been explored.

Taken together, this manuscript contributes to the recent literature focusing on analyzing the influence of LMX on individual creativity by testing a moderated mediation model and illuminating the process model linking LMX to individual creativity [4,13,21,25].

## 2. Theoretical Background

### 2.1. LMX, Engagement, and Creativity 

Drawing on role theory and social exchange theory, researchers have linked LMX to attitudinal and behavioral outcomes [49]. In general, low-quality relationships are orientated towards an economic exchange, while high-quality relationships, focused on longer-term social exchange, are based on high levels of mutual obligations, trust, respect, and reciprocity [49,50,51].

It has been argued that work interactions characterized by mutual appreciation, support, respect, and trust promote engagement because they are a source of meaning and safety for employees [26,29]. Shuck and Wollard [52], based on Kahn’s [26] framework, point out that engagement arises in environments that cultivate psychological meaningfulness and safety, and where employees believe they have the resources to perform their jobs (psychological availability) [26,28]. Psychological meaningfulness is defined as a “sense of return on investments of self in role performance” ([26], p. 705). Psychological safety is conceptualized as a “sense of being able to show and employ self without fear of negative consequences to self-image, status or career” ([26], p. 705) and psychological availability implies to have resources necessary for investing self in role performances.

According to Kahn [28], a sense of meaning emerges “when we are treated with a certain amount of dignity, respect and appreciation by others with whom we work—particularly by supervisors” (p. 24). Under conditions of high-quality LMX, employees feel important, valuable, and valued by their supervisors [26]; they perceive that they can express themselves in their role performance, openly share their ideas, and experiment without fear of negative consequences following failure [26,53]. Employees will be willing to invest all their energies (physical, emotional, and cognitive) into their work roles if they believe that they possess the physical and socio-emotional resources to fulfil the work role demands [26,27]. In a high-quality LMX relationship, it is assumed that followers have access to relevant resources (information, empowerment, feedback, recognition, dignity, emotional support, etc.). Indeed, Breevaart et al. [54] provided evidence linking high-quality LMX to more a resourceful work environment; employees reported more developmental opportunities and social support, which facilitated their engagement. 

Based on social exchange theory [22], Cheng et al. [55] argued that “engagement is a form of currency in the social exchange and a means of fulfilling obligations for reciprocity” (p. 87). In a sample of employees from different industries in Taiwan, the authors found that employees’ perceptions about the quality of the relationship with their supervisors were directly and positively linked to their engagement level. The studies by Agarwal et al. [37], on a sample of managerial employees in India, and Brunetto et al. [56], on a sample of nurses and police officers in Australia, also support this relationship. Additionally, the meta-analysis by Christian et al. [36] revealed a positive estimated corrected correlation between LMX and engagement. According to the previous arguments, it is posited that

**Hypothesis** **1.** *LMX is positively related to employee engagement.*

According to the creativity componential theory [1,14], while domain-relevant skills and creativity-relevant skills determine individuals’ ability to perform creatively, intrinsic task motivation determines their actual behavior. If individuals are intrinsically motivated, the likelihood of truly engaging in their work and spending more time and energy searching for new information and different solutions in their work role will be increased [57]. Drawing on the creativity componential theory, Bakker and Xanthopoulou [58] stated that an employee “who is not engaged is not going to use his/her skills and expertise in the service of creative performance” (p. 2763). Atwater and Carmeli [3] concluded that “without energy, creative abilities will not be optimized and the employees are less likely to be creatively involved in work” (p. 266).

Furthermore, because activating positive moods improve cognitive processes and stimulate the search and integration of new information and the consideration of multiple alternatives, they seem to be significant for creativity [59,60]. The positive emotions usually felt by engaged employees expand their action-thinking repertoires [61]. This enlargement encourages the building of enduring psychological and intellectual resources, which will boost learning and creativity. 

A few studies have concluded that feelings of energy and vitality are positively linked to creativity [3,62,63]. Although these studies are very informative, engagement differs from feelings of energy and vitality because it encompasses “the investment of an individual’s complete self” ([27], p. 617) in the job. In addition to high activation, engagement denotes how intensely and persistently employees invest all their energies in their work [27]. Cognitive perseverance and persistence contribute to the generation of more ideas and new solutions [60]. Since creativity requires effort, mental energy, persistence, and high employee involvement [3,64], highly engaged employees are likely to exhibit more curiosity, are willing to assume risks, enthusiastically look for new ideas, consider multiple alternatives, and explore unconventional ways and approaches to solve problems and challenges [65]. Employees who bring all their energies to, physically and emotionally, and are cognitively focused on their work will be willing to employ their knowledge, ability, and expertise and to make a greater effort to acquire new skills to be creative [14].

To the best of our knowledge, only two studies have tested the engagement–creativity relationship. Bakker and Xanthopoulou [58] discovered that engaged school principals were considered more creative by teachers. Eldor and Harpaz [48] revealed that engagement promoted employees’ creativity in different occupations in Israel. Accordingly, the following is proposed

**Hypothesis** **2.** *Engagement is positively related to individual creativity.*

The creativity componential theory proposes that social environment factors, such as relationships with supervisors, affect individual creativity but that their effect on motivation is more direct [14]. As previously argued, a positive relational environment creates the favorable psychological conditions (meaningfulness, safety, and availability) for employees to invest and express themselves freely in their jobs, which, in turn, stimulates them to generate and derive new solutions and approaches. Recently, Lee et al. [12] have highlighted that creativity thrives when employees are in psychologically safe and motivating environments. The relational leadership style, and LMX in particular, is considered to be a practice that encourages employees’ engagement [11], so that they direct all their energies towards creativity. Furthermore, according to social exchange theory, employees in high-quality LMX relationships feel obligated to reciprocally respond to the good treatment by their supervisor by showing positive attitudes and behaviors [37,38,66]. Engagement is considered a more immediate way to respond reciprocally to social exchanges [55,67]. Therefore, employees in a high-quality LMX relationship will be more engaged and, consequently, more involved in beneficial behaviors, such as creativity. Highly engaged employees, by bringing their whole self into their work roles performance, will be more involved in beneficial behaviors, such as creativity [48,58,65].

Although no study has tested engagement as a mediating mechanism between LMX and individual creativity, Atwater and Carmeli [3] showed that positive exchange relationships between leaders and subordinates helped employees have the sensation of greater energy and become more involved in creative tasks. Furthermore, Agarwal et al. [37] and Kim and Koo [38] confirmed that engagement is a mediating mechanism between LMX and innovative behavior. Based on the aforementioned arguments, engagement is expected to explain the effect of LMX on individual creativity.

**Hypothesis** **3.** *LMX is indirectly related to individual creativity via engagement.*

### 2.2. Job Complexity as a Moderator

Recently, Morgeson and Humphrey [68] broadened the classical model of job characteristics including additional motivational characteristics related to knowledge. Job complexity, which is defined as “the extent to which a job is multifaceted and difficult to perform” ([40], p. 1335), is increasingly considered as a crucial knowledge characteristic of current jobs with important motivational outcomes [2,68]. Complex jobs are mentally challenging, and employees are expected to discover new strategies for performing them. The perception of challenge can help employees “to invest more efforts to successfully fulfil the demands entailed by the job” ([62], p. 364) and will result in greater creativity. A meta-analysis conducted by Marinova et al. [41] found that high-complexity jobs stimulate change-oriented behaviors. 

Evidently, creativity is a result of the interplay of individual factors and the work context [39]. According to Blumberg and Pringle [69], although the individual has the capacity and willingness to perform a certain behavior, its realization depends on the presence or absence of certain factors in his/her working context. Parker and Griffin [70] posited that “context can moderate both the extent to which an engaged individual performs well and how they express their engagement” (p. 64). The extent to which engagement fosters individual creativity can, therefore, vary depending on task context, represented by the job complexity level. 

High-complexity jobs are multifaceted, less specified [40,43], and offer greater potential for creativity. In high-complexity jobs, employees have more opportunities to explore new ideas, search for novel solutions and approaches, and solve non-routine problems in their work [41,42]. At the same time, complex jobs are more mentally challenging, require the use of diverse advanced skills [43,68] and the development of new skills to address the challenging job demands [45]. Pan et al. [66] found that empowered employees working in organic structures, in which the jobs are complex and challenging, displayed higher levels of creativity than employees working in mechanistic structures.

As aforementioned, engaged employees fully bring themselves to the job; invest their physical, emotional, and cognitive energies simultaneously; persist in the face of challenges [26,27]; and have high learning goal orientation [71]. Therefore, it is expected that highly engaged employees in complex jobs have the opportunity to better capitalize their energies into creative performance. 

In contrast, low-complexity jobs, which involve relatively uncomplicated and simple tasks [40,68], offer limited opportunities for employees to experiment and attempt different ways of performing their work [41]. These jobs provide less room and incentive for employees “to rock the boat and to make the difference with their ideas” ([45], p. 613). In this context, employees are likely to focus their energies on applying accepted knowledge and practices rather than developing new ways of performing their work, discovering different solutions or generating new ideas in their work [45,72]. When jobs are low in complexity, engaged employees, due to context constraints, are supposed to display a lower level of creativity. 

Finally, when jobs are highly complex, but employees are distanced from their work roles, they will probably reduce their creative efforts. Although the job allows for creativity, employees lack the motivation, energy, enthusiasm, and perseverance necessary to face the challenges involved in creative work [42]. When both variables have small values (low-complexity job and low engagement), it is more difficult for creativity to thrive. According to the aforementioned arguments, the following is posited:

**Hypothesis** **4.** *Job complexity moderates the positive relationship between engagement and individual creativity.*

Since a satisfactory exchange relationship, characterized by trust, appreciation, and positive feedback generates in the followers a sense of psychological meaningfulness and security, and provides them with access to necessary resources [26,29], they will feel more energized and connected to their jobs [37,56]. However, engaged employees may vary in the degree to which they involve themselves in creative behaviors. Jobs vary in their degree of complexity. As noted above, complex jobs are more mentally stimulating, less structured, and generate more opportunities for their occupants to use their knowledge and skills in problem solving [40], providing them more space for creativity. Therefore, based on the interactionist perspective [39], which assumes that individual creativity is the result of the interaction between individual and contextual factors, job complexity may shape the extent to which employees express their engagement (as a result of LMX) in their creativity performance. 

We propose that a high-quality relationship between leader and subordinates will be always beneficial for employees’ engagement; however, if the task context provides them with the opportunity to discover new solutions and apply their potential (perception of job complexity), their engagement will be translated into greater creativity. In sum, we posit a moderated mediation model for individual creativity [73].

**Hypothesis** **5.** *The positive indirect influence of LMX on individual creativity, via engagement, is stronger when job complexity is higher.*

**Figure 1:**
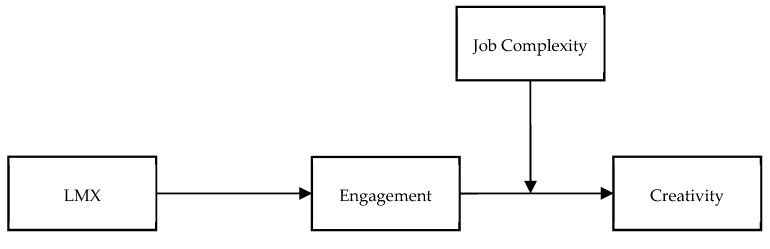
Hypothesized model.

The hypothesized model is shown in Figure 1.

## 3. Method

### 3.1. Sampling Procedure and Participants

The data were gathered from a sample of employees from emergent high-tech and knowledge-based SMEs in Spain. Various sources of information were used to select the firms in which to carry out the study: the list of firms linked to the Spanish universities associated with RedEmprendia (a network of universities in Spain, Portugal, and Latin America that promote responsible innovation and entrepreneurship), the spin-off registers of the websites of the main Spanish universities and those of firms in the main technology parks in Spain. From the resulting list, we contacted, by mail or phone, with the management of 100 firms that satisfied three conditions: SMEs, with an age less than 10 years, and belonging to high-tech and knowledge-intensive industries. Of these, 21 agreed to collaborate in the investigation. According to Messersmith and Guthrie [74], emergent firms “are the foundation for economic growth in today’s business” (p. 241). High-tech and knowledge-based SMEs need the employees to develop fresh ideas and explore unconventional solutions as a basis for developing advanced processes and launching new products and services to the market [6].

To collect the data, and at the same time guarantee the respondents’ anonymity, we mailed employees a direct link to the online questionnaire. Specifically, we contacted the 443 employees whose emails were provided to us by the participating firms. Finally, we received questionnaires of 320. Males constituted 63.75% of the sample, and female accounted for 36.25%. The respondents had a mean age of 32.09 years. The sample consists of employees with a high educational level. The majority had a higher education degree (69.06%); 27.81% had a master’s degree, or PhD; and only 3.12% had high school or lower level studies.

### 3.2. Measures

Except for the control variables, we measured all the constructs with multiple items using a seven-point Likert scale (1 = strongly disagree, 7 = strongly agree). Every measure was taken or adapted from previously validated and published instruments. All measurement items are available in the Appendix A.

LMX: We used the eight-item scale proposed by Bernerth et al. [50]. An example is “My relationship with my manager is composed of comparable exchanges of giving and taking”. 

Engagement: We used 12 items from Rich et al.’s [27] three-dimension scale: physical (e.g., “I try my hardest to perform well on my job”), emotional (e.g., “I am enthusiastic in my job”), and cognitive (e.g., “At work, I focus a great deal of attention on my job”). According to Rich et al. [27], since job engagement is reflected by the commonality of the three dimensions, it is a second order construct. This measure, based on the Kahn’s [26] three-factor conceptualization, emphasizes the motivational nature of engagement. Therefore, it is especially suitable for analyzing the effect of engagement on the individual creativity. 

Job complexity: We used three items from Morgeson and Humphrey’s [68] scale. To improve the participants’ understanding, the items were worded positively. An example item is “The job comprises relatively complicated tasks”. 

Individual creativity: We took the four-item scale used by Baer and Oldham [65]. An example is “I often come up with creative solutions to problems at work”. 

Employees’ age, gender (0 = male, 1 = female), education level (1 = vocational training; 6 = PhD), and organizational tenure were introduced as control variables since they could have effects on engagement and creativity [3,25,46,48,65].

### 3.3. Analytical Methods

Statistical analyses were conducted with SPSS Statistics 24.0 and SPSS AMOS 23 (IBM Corp, Armonk, NY, USA). To establish the psychometric properties of the scales, confirmatory factor analyses (CFAs) in AMOS were conducted. To test the hypotheses, the PROCESS macro for SPSS [73] was applied with bias-corrected bootstrapping. Bootstrapping does not presume that data are normally distributed, so this statistical procedure is recommended to validate mediating effects. Moreover, the PROCESS macro for SPSS incorporates conditional process analysis. According to Hayes [73], this methodology “focuses on the estimation and interpretation of the conditional nature (the moderation component) of the indirect and/or direct effects (the mediation component) of X on Y in a causal system” (p. 11). Therefore, the PROCESS macro is particularly adequate to analyze the proposed model. To enhance the interpretability of the coefficients, the variables implicated in the interaction were mean-centered [73].

## 4. Results

As displayed in Table 1, the correlations were aligned with our hypotheses. To confirm the constructs’ reliability and validity, we conducted CFAs. First, we analyzed the engagement measure fit as a second-order factor formed by three dimensions. The results indicated a satisfactory fit (χ2 (49) = 119.60; χ2/df = 2.44; CFI = 0.98; TLI = 0.97; RMSEA = 0.07). Second, we tested the fit of the structural model. The results revealed an adequate fit with the data (χ2 (316) = 870.53; χ2/df = 2.76; CFI = 0.93; TLI = 0.93; RMSEA = 0.07), implying the acceptability of the proposed model.

Table 2 summarizes the statistics used to verify the scales’ reliability and validity. The composite reliability (CR) and the average variance extracted (AVE) of each scale exceeded the established minimum of 0.6 and 0.5, respectively [75], supporting the reliability of the scales. Convergent validity was evidenced by verifying that all the factor loadings were significant and higher than 0.5. The discriminant validity between constructs was verified since the correlation confidence intervals excluded the unit value, and their squared correlations were lower than the AVE [75].

The presence of a mediating effect requires that two conditions be fulfilled [76]: the independent variable (LMX) should be significantly associated with the mediator (engagement), and the mediator should be significantly related with the dependent variable (individual creativity). To test the hypotheses, Model 14 established by Hayes [73] was performed. Table 3 presents the results for the conditional process model. In support of hypothesis 1, LMX was found to be positively linked with engagement (a = 0.22, *p* < 0.001). Consistent with hypothesis 2, engagement had a positive effect on individual creativity (b1 = 0.55, *p* < 0.001). The validation of the two first hypotheses provides evidence for the indirect influence of LMX on individual creativity via engagement, confirming hypothesis 3. Moreover, confidence intervals for the indirect conditional effects of LMX on individual creativity at various values of job complexity estimated by bootstrap did not include zero (Table 4). This provides additional evidence supporting hypothesis 3.

**Figure 2:**
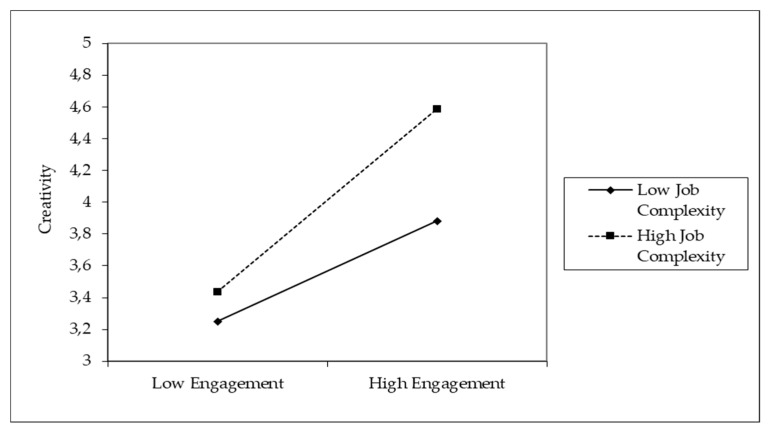
Moderating effect of job complexity on the engagement–creativity relationship.

The interaction effect between engagement and job complexity was positive and significant (b3 = 0.13, *p* < 0.05), confirming hypothesis 4. Figure 2, plotted using the process by Dawson [77], supports this interpretation of the moderating effect by showing that the engagement–individual creativity relationship is significantly strongest when job complexity is higher.

As seen in Table 4, the indirect influence of LMX on individual creativity via engagement is moderated by job complexity (hypothesis 5). Since the confidence interval estimated by the bootstrap for the index of moderated mediation excludes zero (0.003–0.061), this result provides additional support for hypothesis 5 [78]. It was evidenced that the higher the job complexity the stronger the indirect influence of LMX on employee creativity was. 

## 5. Discussion

### 5.1. Results Discussion 

The purpose of this investigation was to advance the understanding of the relationship between LMX and individual creativity. In particular, this paper explored how the quality of LMX, as perceived by followers, was associated with individual creativity through engagement and how this relationship was moderated by job complexity in the context of high-tech and knowledge-based SMEs. The findings of this research supported the proposed framework. The results indicated that (1) the quality of the relationship between leader and follower favors job engagement; (2) engagement, in turn, increases individual creativity. Finally, the results showed that job complexity interacts with engagement to increase individual creativity. In other words, the greater the job complexity, the greater the effect of engagement on individual creativity. 

Unlike other studies (e.g., Atwater and Carmeli [3]; Pan et al. [66]; Qu et al. [25]), our study found no direct relationship between LMX and individual creativity; that is, LMX did not directly increase employee creativity. However, an indirect relationship was found. The implication is that the quality of LMX encourages employees to be willing to invest all their energies into their work and display their real identity, thoughts, and feelings [26]; and through this motivational mechanism, LMX boosts employees’ creativity. This result is aligned with earlier research [37,55,56] and engagement literature [26,29]. High-quality exchanges cultivate employee engagement probably because they deepen the sense of meaning about the purpose of the work [29] and encourage a sense of safety by embodying the supportive regard and trusting interactions necessary for employees to invest and express themselves in their role performances [26]. In turn, engagement impacts individual creativity, which is an important outcome for high-tech and knowledge-based SMEs [6]. This result is consistent with the notion that motivation is fundamental for creativity [1,14,57] and research concerning activating positive mood states. Engaged employees experience high activating positive emotions (e.g., enthusiasm, excitement, and interest) and have a broader vision of their work role [27]. Therefore, they are more likely “to think outside the box” ([48], p. 217) and produce novel ideas and different solutions.

This finding is also consistent with the Job Demands–Resources model of work engagement [79], according to which resources (namely, LMX), by activating the motivational process leading to work engagement, impact on employee behaviors. For example, Bakker and Bal [80] found that the weekly exchange with the supervisor was associated with weekly engagement which, in turn, led to weekly job performance.

Furthermore, it was found that the interaction between engagement and job complexity promotes individual creativity. Job complexity accentuates the impact of engagement on individual creativity. The highest levels of creativity were reached when both engagement and job complexity were high. When a job provides little room for personal contribution and is undemanding and not challenging (low-complexity job), an engaged employee has less of a chance to integrate diverse information and come up with unusual and singular ideas while working. In line with that proposed by Cai et al. [11], an synergistic effect of job design (contextual factor) and job engagement (individual factor) for predicting individual creativity was found.

Additionally, it was confirmed that the intensity of the mediated relationship (LMX–engagement–individual creativity) is contingent on job complexity. The indirect influence of LMX (via engagement) on individual creativity was stronger when jobs were more complex. This implies that a good exchange relationship between superior and employee encourages his/her engagement level; but for this to be translated into elevated creativity, the employee must have the opportunity to contribute creatively, and a high-complexity job affords such an opportunity to a greater extent. Moreover, it is likely that in complex jobs engaged employees (due to the greater self-regulation) will be more willing to apply their talents and cultivate new skills to produce novel and useful ideas.

### 5.2. Theoretical Contributions

This study has several theoretical contributions. By proposing and testing a model of creativity that integrates social context (LMX), motivational (engagement), and task context (job complexity) variables, this study addresses several gaps identified in the recent literature. First, by finding empirical evidence for the mediating role of engagement, which is a robust motivational construct [27], this study provides a clearer understanding of the process model linking LMX to individual creativity, thereby contributing to enrich the existing LMX and creativity literature. In this way, we respond to calls for new studies that analyze the mediating variables in the LMX–individual creativity relationship (e.g., Khalili [4]; Qu et al. [25]). To the best of our knowledge, this is the first research to test the LMX–engagement–individual creativity relationship.

Second, it contributes to fill the knowledge gap in research regarding the role of motivational psychological states on creativity [2]. Although engagement has been related to individual outcomes such as personal initiative [81], active learning [82], or knowledge sharing [83], little is known about its effects on individual creativity.

Finally, this research responds to the calls made by earlier studies [13,25,47,54] in examining the moderating role of job complexity in the engagement–creativity relationship. The results also support Parker and Griffin’s [70] notion that an employee may exhibit a lower level of performance (creativity in our study) not because he/she is not highly engaged but because the context (job complexity in our study) limits that possibility. At the same time, we extend the research by Volmer et al. [19], who found that LMX contributed more strongly to followers’ creativity when they experienced higher job autonomy. As expected, when employees in high-quality LMX relationships perform complex tasks, they capitalize their greater engagement on increasing their creativity. We think this finding represents an important advance in the comprehension of the mechanisms involved in the complex relationship of LMX–creativity.

### 5.3. Practical Contributions

The findings of this study provide several practical implications for management at high-tech and knowledge-intensive SMEs where the knowledge and creativity of the personnel is a major resource [9]. 

First, we found that employee engagement is key to encourage individual creativity. This means that when employees are engaged, they are more likely to provide new and useful ideas that will contribute to the success of the firm. In today’s changing environment, especially in the study sector, having employees who provide new and fresh ideas is of paramount importance to firms [5,6,13]. Therefore, management should facilitate employee engagement. 

Second, although there are different ways to increase employee engagement (for example, providing organizational support), our study corroborated the important role of leader–follower relationships. If the relationship is based on trust, respect, and reciprocity, employees will feel more secure, recognized, and valued by their leaders [26,29]. In a high-quality LMX, the leader facilitates the follower to access a substantial amount of support, information, and resources; in turn, the follower is willing to invest more of him/herself in the work role [54]. Although an exchange relationship involves both the leader and the follower, the leader is often responsible for initiating and maintaining the relationship [84]. Accordingly, supervisors must be encouraged to build and nurture high-quality relationships with followers. Management should provide supervisors with the training and resources needed [47]. In this sense, training in interpersonal skills and emotional intelligence becomes relevant. It has been pointed out that leaders with higher emotional intelligence skills, who are able to recognize and understand the emotions of their followers and regulate emotions, build higher quality interpersonal relationships with their employees [85]. Previous studies [86,87] show the effectiveness of training programs in improving the quality of the LMX relationships by developing leaders’ interpersonal and active listening skills and spending time getting to know the concerns and work expectations of subordinates.

Third, the findings show that job complexity allows for the maximization of the effect of engagement on individual creativity. Therefore, if high creativity is a desirable result, as is usually the case in high-tech and knowledge-based SMEs, the job design must facilitate creativity [43]. It is advisable to design jobs so that they are perceived by employees as mentally stimulating and challenging, allow them to use their cognitive skills and provide them opportunities for learning, exploring, and experimenting. However, when it comes to designing or redesigning jobs there may be limits to the complexity level. When jobs are already highly complex, “increasing information processing or task variety may produce job overload” ([68], p. 1334). Therefore, we believe that the design of multifaceted jobs should be accompanied by interventions aimed at improving employee engagement, so that synergistic effects are generated. 

### 5.4. Limitations and Future Research Lines

We cannot ignore the limitations of the study. First, the cross-sectional design limits causal inferences. Additional studies with longitudinal data are needed to support the causality of the relations. 

Second, this study used employee self-reports, which may cause common method bias. Although this bias is rarely severe enough to jeopardize the validity of the results [88], the recommendations of Podsakoff et al. [89] concerning the design of questionnaire were followed to reduce this bias. Additionally, Harman’s one-factor test using a CFA [89] was applied. The model in which every item of the four variables loaded onto the same factor fitted data poorly (χ2(324) = 5859.64; χ2/df = 18.09; CFI = 0.34; TLI = 0.29; RMSEA = 0.23), suggesting that common method bias was not a significant issue in our investigation. 

Third, the sample consisted of employees from emergent high-tech and knowledge-intensive SMEs in Spain. Given that only a limited proportion of SMEs operate in these industries, new studies should replicate the conclusions in other industries and contexts. The characteristics of the sample, made up of highly educated employees, should also be considered. This might restrict the generalizability of the results.

## 6. Conclusions

Although the literature has recognized that LMX is linked to individual creativity, it is necessary to go deeper into the mechanisms underlying this relationship and the conditions under which it occurs. A model incorporating engagement as mediating variable and job complexity as moderating variable has been proposed. This model was tested in a context where creativity constitutes a main basis for the innovation process: high-tech and knowledge-intensive SMEs. The findings revealed that engagement transmits the effect of LMX on individual creativity, and that job complexity intensifies the effect of engagement on individual creativity. Moreover, it was confirmed that the strength of the mediated relationship is moderated by job complexity. When employees in high-quality LMX relationships perform complex tasks, they capitalize their greater engagement on increasing their creativity.

## Figures and Tables

**Table 1:** Descriptive statistics, correlations, and reliability coefficients.

Variable	M	SD	1	2	3	4	5	6	7	8
1 Age	32.09	6.18								
2 Gender	-	-	−0.09							
3 Education	4.10	0.88	0.20 ***	0.03						
4 Tenure	3.17	2.53	0.37 ***	−0.19 **	0.03					
5 LMX	5.02	1.44	−0.18 **	−0.10	−0.09	−0.09	(0.97)			
6 Engagement	5.77	0.82	0.04	0.11	−0.01	−0.10	0.35 ***	(0.93)		
7 Job complexity	4.81	1.17	0.12 *	−0.24 ***	0.13 *	0.13 *	0.14 *	0.29 ***	(0.93)	
8 Creativity	4.85	1.19	0.18 **	−0.09	0.04	0.12 *	0.07	0.37 ***	0.32 ***	(0.91)

Notes: *n* = 320. * *p* < 0.05, ** *p* < 0.01, *** *p* < 0.001. M, mean; SD, standard deviation. Values in parentheses are Cronbach’s alphas.

**Table 2:** Overall reliability and validity of the constructs.

	LMX	Engagement	Job Complexity	Creativity
LMX	CR = 0.97			
	AVE = 0.78			
Engagement	SC = 0.09	CR = 0.85		
	(0.20; 0.42)	AVE = 0.66		
Job complexity	SC = 0.02	SC = 0.09	CR = 0.93	
	(0.02; 0.27)	(0.20; 0.42)	AVE = 0.82	
Creativity	SC = 0.01	SC = 0.16	SC = 0.11	CR = 0.91
	(−0.05; 0.20)	(0.27; 0.52)	(0.23; 0.44)	AVE = 0.72

Notes: AVE, average variance extracted; CR, composite reliability; SC, squared correlation.

**Table 3:** Coefficients for the conditional process model.

	Model 1 Engagement					Model 2 Creativity				
Variables	Coeff	SE	*p*	LLCI	ULCI	Coeff	SE	*p*	LLCI	ULCI
Constant	−3.40	0.88	<0.001	−5.13	−1.66	2.63	1.27	0.04	0.12	5.13
Age	0.67	0.25	0.01	0.17	1.17	0.72	0.36	0.05	0.01	1.43
Gender	0.25	0.09	0.01	0.07	0.43	−0.17	0.13	0.21	−0.43	0.10
Education	−0.00	0.05	0.98	−0.10	0.10	−0.02	0.07	0.77	−0.16	0.12
Tenure	−0.03	0.02	0.12	−0.06	0.01	0.03	0.03	0.22	−0.02	0.08
LMX	0.22	0.03	<0.001	0.16	0.28	−0.05	0.05	0.26	−0.14	0.04
Engagement						0.55	0.09	<0.001	0.38	0.72
Job complexity						0.18	0.06	0.001	0.07	0.30
Engagement X Job complexity						0.13	0.06	0.03	0.01	0.25
R^2^		0.16					0.23			
*F*		12.20					11.50			
*p* value		<0.001					<0.001			

Notes: *n* = 320; bootstrap sample size = 5000; CI = 95%. Coeff, coefficients; SE, standard error; *p*, *p*-value; LLCI and ULCI, lower and upper levels of confidence interval.

**Table 4:** Conditional indirect effects of leader–member exchange (LMX), via engagement, on creativity at different values of job complexity.

Job Complexity *	Indirect Effect	Boot SE	Boot LLCI	Boot ULCI
3.00	0.07	0.03	0.01	0.14
4.00	0.10	0.02	0.05	0.15
5.00	0.13	0.03	0.08	0.19
5.67	0.14	0.03	0.09	0.22
6.00	0.15	0.04	0.10	0.24

∗ Values are for the 10th, 25th, 50th, 75th, and 90th percentiles. Notes: *n* = 320; bootstrap sample size = 5000. Boot SE, bootstrap standard error; Boot LLCI and Boot ULCI, bootstrap lower and upper levels of confidence interval.

## Appendix A

### Appendix A.1. Items Measuring LMX

1. My manager and I have a two-way exchange relationship.
2. I do not have to specify the exact conditions to know my manager will return a favor.
3. If I do something for my manager, he or she will eventually repay me.
4. I have a balance of inputs and outputs with my manager.
5. My efforts are reciprocated by my manager.
6. My relationship with my manager is composed of comparable exchanges of giving and taking.
7. When I give effort at work, my manager will return it.
8. Voluntary actions on my part will be returned in some way by my manager.

### Appendix A.2. Items Measuring Engagement

*Psychical dimension*

1. I exert my full effort to my job.
2. I devote a lot of energy to my job.
3. I try my hardest to perform well on my job.
4. I strive as hard as I can to complete my job.

*Emotional dimension*

1. I am enthusiastic in my job.
2. I feel energetic at my job.
3. I am interested in my job.
4. I feel positive about my job.

*Cognitive dimension*

1. At work, my mind is focused on my job.
2. At work, I pay a lot of attention to my job.
3. At work, I focus a great deal of attention on my job.
4. At work, I am absorbed by my job.

### Appendix A.3. Items Measuring Job Complexity

1. The tasks on the job are difficult and complicated.
2. The job comprises relatively complicated tasks.
3. The job involves performing relatively difficult tasks.

### Appendix A.4. Items Measuring Individual Creativity

1. I suggest many creative ideas that might improve working conditions at my organization.
2. I often come up with creative solutions to problems at work.
3. I suggest new ways of performing work tasks.
4. I am a good source of creative ideas.
